# SAMD1 attenuates antiphospholipid syndrome‐induced pregnancy complications

**DOI:** 10.1002/iid3.1006

**Published:** 2023-10-30

**Authors:** Ran An, Yanqi Yang, Lei Liu, Peiling Li

**Affiliations:** ^1^ Department of Obstetrics and Gynecology The Fourth Affiliated Hospital of Harbin Medical University Harbin Heilongjiang P.R. China; ^2^ Department of Obstetrics and Gynecology The Second Affiliated Hospital of Harbin Medical University Harbin Heilongjiang P.R. China

**Keywords:** antiphospholipid syndrome, embryo loss, SAMD1, vascular injury

## Abstract

**Objective:**

This study was intended to investigate the effect of SAMD1 on antiphospholipid syndrome (APS)‐induced pregnancy complications in mice.

**Methods:**

The mRNA and protein expression of SAMD1 in APS patients and healthy controls was detected by qRT‐PCR and western blot. Anti‐B_2_GPI and ACA levels were tested by ELISA, MMP‐9, iNOS, ICAM‐1 and MCP‐1 mRNA and protein levels determined by qRT‐PCR and western blot, cellular senescence detected by β‐galactosidase staining, cell proliferation ability detected by CCK‐8 assay, cell viability detected by trypan blue staining, cell mobility detected by Transwell, and cell angiogenesis ability detected by matrigel tube formation assay. An APS pregnant mouse model was constructed, and the embryo absorption rate was calculated.

**Results:**

SAMD1 expression was low in serum of APS patients, which was correlated with the history of thrombosis and the number of adverse pregnancies. Anti‐B_2_GPI and ACA levels were increased in APS. The expressions of MMP‐9, iNOS, ICAM‐1, and MCP‐1 were also significantly upregulated in HUVECs treated with APS serum. APS promoted HUVEC senescence and inhibited cell proliferation, migration and angiogenesis. Overexpression of SAMD1 reversed the above results. Experiments on the APS pregnant mouse model confirmed that overexpression of SAMD1 reduced the rate of fetal loss.

**Conclusion:**

SAMD1 may reduce APS‐induced embryo loss by regulating cellular senescence, proliferation, migration, and angiogenesis.

## Graphical abstract

SAMD1 may reduce APS‐induced vascular injury and embryo loss by regulating cellular senescence, proliferation, migration, and angiogenesis.

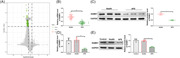

## 1 INTRODUCTION

Antiphospholipid syndrome (APS) is defined as an autoimmune multisystem disease characterized by the development of arterial and venous thromboembolic events and/or pathological pregnancies, mainly recurrent abortion, under the condition of persistently positive antiphospholipid antibody (aPL).^1^, ^2^, ^3^ APS can be primary or occur in patients with systemic lupus erythematosus (SLE) or other systemic autoimmune diseases.^4^ Dysregulation of immune function in APS patients produces a variety of autoantibodies, which cause thrombocytopenia while disrupting the integrity of vascular endothelial cells, leading to thrombosis.^5^ In pregnant women, placental supply is blocked due to thrombosis of the placental arteries and veins, resulting in abortion.^6^ It is estimated that the prevalence of APS is 50/100,000 people, with an incidence of 2.1/100,000 person/years and without significant difference in gender.^7^ aPLs bind to phospholipids and plasma or membrane proteins expressed in endothelial cells, fibroblasts or trophoblasts, making contribution to a pro‐thrombotic state.^8^ Beta‐2‐glycoprotein I (B_2_GPI) is considered as the dominant antigen in APS and anti‐B_2_GPI is used as the laboratory diagnosis criteria for APS.^9^ The treatment of APS mainly includes anticoagulant therapy and antiplatelet therapy, such as aspirin, heparin, and warfarin.^10^, ^11^ Research on molecular mechanism may provide evidence and support for the targeted therapy for APS.

The sterile alpha motif (SAM) domain is a putative protein module reported to exist in eukaryotic genomes, which is involved in building large protein complexes in the cell.^12^ This motif is reported in multiple signaling molecules, including but not restricted to regulators of lipid metabolism, serine/threonine protein kinases, and GTPases, which may play a potential role in the development of eukaryotes.^13^ SAM domain containing 1 (SAMD1) is a repressive chromatin regulator and predicted transcriptional repressor, which is implicated in atherosclerosis through binding with LDL on cell surface and promoting LDL oxidation which contributes to the formation of foam cells.^14^ Patients with SLE or APS are more susceptible to atherosclerotic cardiovascular events; immunological alterations, such as antibodies to oxidized LDL, aPLs, and antibodies to B_2_GPI, may have implications for premature atherosclerosis in SLE and APS.^15^ In addition, SAMD1 has been implicated in the proliferation of hepatocellular carcinoma cells ^16^ and in muscle adaptation.^17^ By far, there is no report on the function of SAMD1 in APS. This study is the first to investigate the link between SAMD1 dysregulation and APS‐induced pregnancy complications. Moreover, the primary mechanism of action of SAMD1 in APS is also determined.

## 2 MATERIALS AND METHODS

### 2.1 Bioinformatics analysis

A data set GSE50395 was downloaded from GEO database (http://www.ncbi.nlm.nih.gov/geo). The differentially expressed genes between APS patients and healthy controls were analyzed using R language limma package with |logFC|>1 and adjust *p* < .05 as standard screening criteria.

### 2.2 Case selection and serum preparation

A total of 58 patients who visited the obstetrics and gynecology department at the Second Affiliated Hospital of Harbin Medical University due to adverse pregnancy from June 9, 2018 to October 18, 2020 and were diagnosed with APS according to the International Consensus Statement on Preliminary Classification Criteria for APS issued by the Sapporo International Congress in 1999 were selected for this study. During the same period, another 46 healthy female volunteers with no history of adverse pregnancy, no history of thrombosis, and no history of autoimmune diseases were selected as controls. This study acquired consent from the Ethics Committee of the Second Affiliated Hospital of Harbin Medical University (No. 2022‐SCILLSC‐18) and was carried out as required by the Declaration of Helsinki, and all subjects signed an informed consent form for this study. Blood sample (5 mL) was collected from each patient in the morning under fasting condition and centrifuged at 2000*g*. The peripheral blood serum was obtained and stored at −70°C.

### 2.3 Cell culture

The human umbilical vein endothelial cell (HUVEC) line was purchased from BeNa Culture Collection (BNCC). HUVECs were cultured in high‐glucose DMEM (supplemented with 10% FBS and 1% P/S) in a 37°C, 5% CO_2_ incubator. Adherent HUVECs were digested with solution containing 0.25% trypsin and 0.02% EDTA and prepared into cell suspension for passage every 2–3 days.

### 2.4 Group design and cell treatment

Cells were seeded in 6‐well plates and divided into five groups: (1) blank control group (shortened as control): no treatment to the cultured cells; (2) normal control group (shortened as health): 10% peripheral blood serum from healthy controls was added to the cell culture medium; (3) APS serum group (shortened as APS): 10% peripheral blood serum from APS patients was added to the cell culture medium; (4) negative control group (shortened as pcDNA3.1‐NC): cells cultured in medium with 10% peripheral blood serum from APS patients were transfected with pcDNA3.1 plasmid using Lipofectamine 3000 kit (Invitrogen); and (5) SAMD1 overexpression group (shortened as pcDNA3.1‐SAMD1): cells cultured in medium with 10% peripheral blood serum from APS patients were transfected with pcDNA3.1‐SAMD1 plasmid and the effectiveness of transfection was evaluated by qRT‐PCR after 48 h.

### 2.5 qRT‐PCR

Serum and cellular total RNA were extracted using TRIzol (Invitrogen) and reverse transcription was carried out with a reverse transcription kit (Takara). Expression of genes was detected using LightCycler 480 quantitative fluorescence PCR instrument (Roche) per the instructions of the quantitative fluorescence PCR kit (SYBR Green Mix), with GAPDH as the internal reference. The specific reaction conditions were: predenaturation at 95°C for 5 min, denaturation at 95°C for 10 s, annealing at 56°C for 10 s, and extension at 72°C for 20 s, with SAMD1, MMP‐9, iNOS, ICAM‐1, and MCP‐1 of 32 cycles and GAPDH of 28 cycles. Data was analyzed using the $2^{–\Delta \Delta C_{t}}$ method, in which $2^{–\Delta \Delta C_{t}}$ indicated the fold relationship of target gene expression between the experimental group and the control group per the following formula: ΔΔ*C*_T_ = Δ*C*_t_ experimental group—Δ*C*_t_ control group, where Δ*C*_t_ = *C*_t_ target gene—*C*_t_ internal reference gene. *C*_t_ was the number of amplification cycles required for the real‐time fluorescence intensity of reaction to reach the threshold, and the amplification was in logarithmic phase growth. The experiment was repeated three times. The amplification primer sequences of each gene and its internal reference were detailed in Table 1.

**Table 1:** Primer sequence.

Name of primer	Sequences
SAMD1‐F	5′‐ACACATCTGCTCCTCAGCAC‐3'
SAMD1‐R	5′‐GAACCCCAACCCAGCTAGTC‐3'
MMP‐9‐F	5′‐ACGGCAGAGAGCATTGTGTA‐3'
MMP‐9‐R	5′‐CCTGTAGCGTAAGAGCCAGAG‐3'
iNOS‐F	5′‐CTATGCTGGCTACCAGTAGC‐3'
iNOS‐R	5′‐CCATGATGGTCACATTCTGC‐3'
ICAM‐1‐F	5′‐CAAGGTGACGCTGAATGG‐3'
ICAM‐1‐R	5′‐CATCGTCGGCGTCAGTATT‐3'
MCP‐1‐F	5′‐GTGTTCAAGTCTTCGGAGTT‐3'
MCP‐1‐R	5′‐CAATAGGAAGATCTCAGTGC‐3'
GAPDH‐F	5′‐AACGGATTTGGTCGTATTG‐3'
GAPDH‐R	5′‐TTAGGGTAGTGGTAGAAGG‐3'

*Note*: F, forward primer; R, reverse primer.

### 2.6 Western blot

Total protein was extracted using RIPA lysis buffer containing PMSF on ice for 30 min. The lysate was centrifuged at 4°C, 8000*g* for 10 min, and then the supernatant was collected. Total protein concentration was tested with a BCA kit. Protein (50 μg) was dissolved in 2 × SDS loading buffer, boiled at 100°C for 5 min, and subjected to SDS‐polyacrylamide gel electrophoresis. The protein was transferred to a PVDF membrane by wet transfer and 5% skimmed milk was used for blocking (room temperature, 1 h). Then the PVDF membrane was incubated with diluted primary antibodies against SAMD1 (PA5‐65308, 1:1000; Thermo Fisher Scientific), MMP‐9 (ab137867, 1:1000; Abcam), iNOS (ab178945, 1:1000; Abcam), ICAM‐1 (ab109361, 1:1000; Abcam), MCP‐1 (ab9669, 1:1000; Abcam) and GAPDH (ab8245, 1:2000; Abcam) overnight at 4°C, washed, and incubated with horseradish peroxidase‐labeled secondary antibody goat antirabbit IgG (1:5000; Beijing ComWin Biotech Co., Ltd) for 2 h at room temperature. Electrochemiluminescence (ECL) was used for color development and finally analyzed on a gel imager. The gray level of the bands was analyzed using Image J software. The experiment was repeated three times.

### 2.7 Enzyme‐linked immunosorbent assay (ELISA)

ELISA was performed for the detection of anti‐B_2_GPI, anticardiolipin antibody (ACA), MMP‐9, iNOS, ICAM‐1, and MCP‐1 levels in serum per the kit instruction (Shanghai Beyotime Biotechnology Co., Ltd). Samples to be tested in each group were taken and added into the reaction wells (100 μL/well), with four duplicate wells for each group, and incubated at 37°C for 90 min, after which the liquid in the wells was discarded. Then the samples were incubated with 100 μL of biotinylated antibody working solution (37°C, 60 min), after which the liquid in the wells was discarded. After washing for three times, the samples were incubated with 100 μL of enzyme conjugate working solution (37°C, 30 min), after which the liquid in the wells was discarded. After washing for five times, the samples were incubated with substrate solution (90 μL per well) at 37°C for 15 min, followed by addition of 50 μL of stop solution. The absorbance (OD value) of anti‐B_2_GPI and ACA in each group of samples at the wavelength of 450 nm was measured. The experiment was repeated three times.

### 2.8 Senescence‐associated β‐galactosidase (SA‐β‐gal) staining

The experiment was performed per the instructions of a β‐galactosidase kit (Shanghai Beyotime Biotechnology Co., Ltd). Cells were cultured in 96‐well plates for 48 h in groups, washed with PBS, and fixed at room temperature for 20 min with 1 mL of fixative. After PBS washing, incubation of cells was performed in 1 mL of staining working solution (staining solution A: 10 μL, staining solution B: 10 μL, staining solution C: 930 μL, and X‐Gal: 50 μL) in a noncarbon dioxide incubator at 37°C overnight. The cells were washed twice with PBS and after 2 mL of PBS was added, three different areas were randomly selected from each well and imaged by a ×100 inverted microscope. The degree of cellular senescence was expressed as the positive rate of cells [(number of blue cells/number of total cells) × 100%]. The experiment was repeated three times.

### 2.9 CCK‐8 cell proliferation assay

HUVECs in each group transfected stably were digested with 0.25% trypsin, seeded in 96‐well plates at 100 μL of 5 × 10^4^ cells/well, with four duplicate wells for each group, and then cultured for 6 h to allow the cells to adhere. CCK‐8 reagent (10 μL) was added at 0, 24, 48, and 72 h after cell attachment, respectively, and the change of the medium color was observed after 1 h. The absorbance was measured at a wavelength of 450 nm with a microplate reader after 3 h. The experiment was repeated three times.

### 2.10 Trypan blue staining

Adherent cells were digested with trypsin after cell suspension was removed, and 1 mL of culture medium was mixed with the cells to prepare single cell suspension. Cells (9 μL) were mixed with 0.4% trypan blue solution (1 μL) at a ratio of 9:1, with a final concentration of 0.04%. Viable and dead cells were counted separately after 3–5 min of staining. Microscopically, dead cells were stained blue, while living cells were colorless and transparent, with the cell viability calculated: viable cell rate (%) = total viable cells/(total viable cells + total dead cells) × 100%. The experiment was repeated three times.

### 2.11 Transwell

HUVECs from each group were resuspended with DMEM free of serum, diluted to 5000 cells/200 μL, and seeded into transwell insert (200 μL). The transwell insert was placed in a 24‐well plate, and 600 μL of endothelial cell complete medium was added to each well to immerse the bottom of the insert. After the 24‐well plate was placed in the incubator for 24 h, the insert was removed and washed with PBS. After the cells were fixed using 4% paraformaldehyde for 30 min, the insert was dried and the bottom was stained with 0.1% crystal violet for 15 min. Cells on the membrane surface at the bottom of the upper chamber were wiped off with a wet cotton swab. After washing with PBS and drying, three fields were randomly selected under a ×100 inverted microscope and the number of migrating cells in each field was counted. The experiment was repeated three times.

### 2.12 Matrigel tube formation assay

Before experiments, 48‐well plates (Millipore), matrigel (Corning), and pipette tips were precooled or dissolved at 4°C. Matrigel (100 μL) was pipetted into a 48‐well plate with a precooled tip, and the plate was put in an incubator (37°C, 30 min). HUVECs (2 × 10^4^) with different treatments were seeded into each well. After 6 h of cell culture, photographs were taken and vessel length was analyzed by Image J software (NIH).

### 2.13 Establishment of pregnant APS mouse model

Eighteen SPF grade female BALB/c mice and 18 male BALB/c mice (6‐week‐old adult mice, weighting 19 ± 3 g) were purchased from the Shanghai Laboratory Animal Center of the Chinese Academy of Sciences. All experimental animals were housed in a SPF‐grade sterile laminar flow room under constant temperature (22°C–26°C) and constant humidity (55% ± 5%). All animal experiments complied with the rules and regulations for laboratory animal management as well as the ethical requirements related to laboratory animals, which were conducted under the approval of the Ethics Committee of the Second Affiliated Hospital of Harbin Medical University.

As previous methods described,^18^ female mice were randomly divided into normal control group (*n* = 6) and APS experimental group (*n* = 12) after 5 days of adaptive feeding. In the APS experimental group, 4 mg of purified human B_2_GPI (50 μg/mouse) was dissolved in 16 mL of PBS and emulsified with 20 mL of complete Freund's adjuvant (CFA) followed by 0.5 mL of subcutaneous injection into female BALB/c mice at three points in the axilla and back. Then the animals were administrated with the same dose of B_2_GPI (4 mg) mixed in PBS and emulsified with incomplete Freund's adjuvant (IFA) every 3 weeks for booster immunization. The mice in the normal control group received PBS/CFA for immunization, which was repeated three times. Serum was separated from normal control group and APS experimental group 3 weeks after the last immunization, and the anti‐B_2_GPI titer was detected by ELISA to verify the model. Female mice in each group cohabited with male BALB/c mice at a ratio of 1:1, and those who were found to have vaginal plugs or sperm on vaginal smears in the next morning were regarded as pregnant at Day 0. Twelve APS mice were randomly divided into pcDNA3.1‐NC group and pcDNA3.1‐SAMD1 group, with six mice in each group. The pcDNA3.1‐NC group and pcDNA3.1‐SAMD1 group were injected with unloaded virus and SAMD1 overexpressing lentivirus via the caudal vein,^19^ respectively, once daily for 15 days. Blood samples were collected from the eye fundus of mice in each group on Day 16. All blood samples were allowed to stand for stratification, placed in a warm water bath, and centrifuged to separate serum. The anti‐B_2_GPI level, ACA level, activated partial thromboplastin time (aPTT), and platelet count (PLT) of each serum sample were measured. Anti‐B_2_GPI and ACA levels were tested by ELISA. The detection of aPTT was performed per the instructions of an aPTT detection kit (Shanghai Long Island Biotech Co., Ltd). PLT was measured by an automatic blood cell analyzer (YYZ‐HF‐3600, Beijing Haifuda Technology Co., Ltd).

### 2.14 Detection of embryo resorption rate

On the 14th day of pregnancy, the mice in each group were anesthetized with urethane for embryo separation. The wet weight of the embryos was weighed with a precision electronic balance. Here, embryonic absorption was defined as no embryos, only residual placental tissue, or a clot in macroscopic appearance. Mice with restricted embryonic growth (i.e., whose body weight was less than one‐third of the body weight of normal pregnant mice) and normal embryonic development were regarded as having no embryonic absorption. The embryo resorption rate was calculated: embryo resorption rate = number of embryos absorbed/(number of embryos absorbed + number of embryos not absorbed) × 100%.

### 2.15 Statistical analysis

The collected raw data were statistically analyzed with GraphPad 7.0 software, and all data were finally expressed as mean ± standard deviation. *T* test was used for comparison between two groups, one‐way analysis of variance for comparison between multiple groups, Tukey's multiple comparisons test for post hoc multiple comparison, and *χ* ^2^ test for analyzing the relationship between SAMD1 expression and pathological characteristics of APS patients. A difference with *p* < .05 had statistical significance.

## 3 RESULTS

### 3.1 Decreased SAMD1 expression in APS

**Figure 1:**
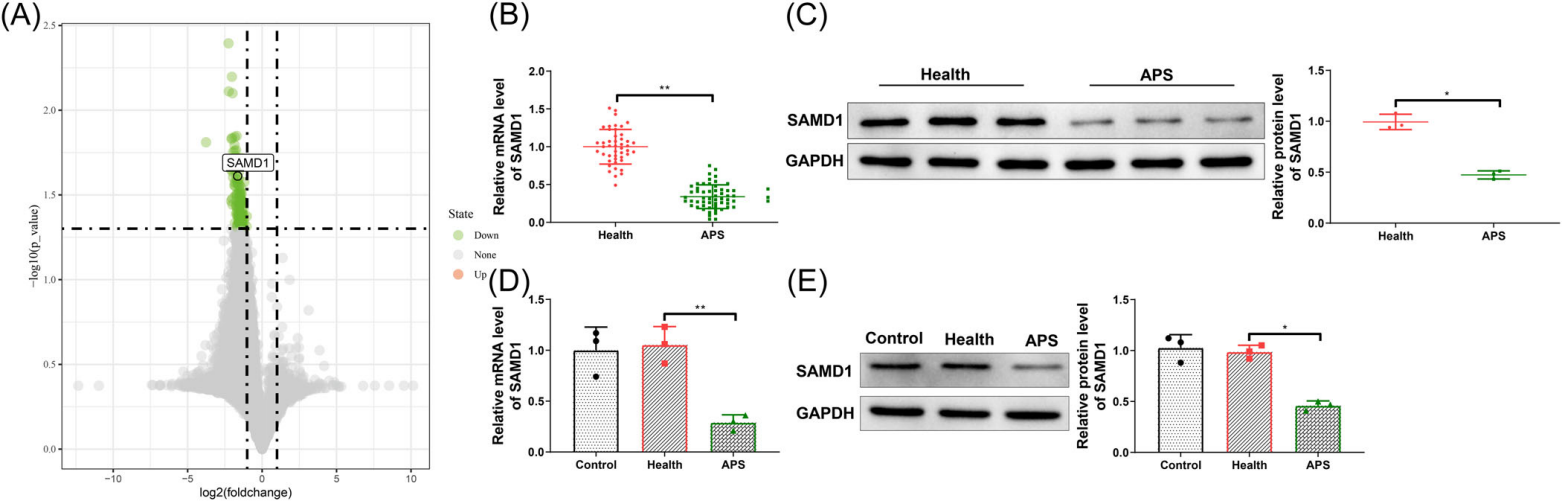
Decreased SAMD1 expression in APS. (A) The expression of SAMD1 in the monocytes of APS patients was decreased compared with healthy controls by GEO database analysis; (B and C) The expression of SAMD1 in the serum of healthy controls and APS patients was detected by qRT‐PCR or western blot; (D and E) The expression of SAMD1 in HUVECs of different groups was detected by qRT‐PCR or western blot. *T* test was used for comparison between two groups, one‐way analysis of variance test for comparisons among multiple groups, and Tukey's multiple comparisons test for post hoc multiple comparisons. *p < .05, **p < .01, ***p < .001. APS, antiphospholipid syndrome.

Based on the GSE50395 data set of the GEO database, we found that SAMD1 expression was decreased in monocytes from APS patients compared with that from healthy control women (Figure 1A). To explore the relationship between SAMD1 and APS, we first detected the expression of SAMD1 by qRT‐PCR in the collected serum samples from 58 APS patients and 46 healthy women, and the results concluded that SAMD1 expression was lower in the serum of APS patients (Figure 1B,C, *p* < .05). The analysis of the correlation between SAMD1 and different clinicopathological characteristics of APS patients showed that SAMD1 expression in the peripheral blood serum of APS patients were correlated with the history of thrombosis and the number of adverse pregnancy deliveries, but not with the patient's age, BMI, and the number of pregnancies (Table 2). We further detected the expression of SAMD1 in HUVECs of the control group (without any treatment), health group (cultured with 10% healthy serum), and APS group (cultured with 10% APS patient serum), and the qRT‐PCR and western blot found that the expression of SAMD1 in the APS group was lower than that in the health group and control group (Figure 1D,E, *p* < .05), but no obvious difference was seen between the health group and the control group. The above findings showed indication that the expression of SAMD1 was downregulated in APS.

**Table 2:** Correlation between SAMD1 expression and clinicopathologic features of APS.

Clinicopathologic features	*N*	SAMD1 expression		*p* Value
		Low (*n* %)	High (*n* %)	
Age (years)				*n.s* > .05
≥35	9	5 (55.6)	4 (44.4)	
30–35	26	14 (53.8)	12 (46.2)	
≤30	23	10 (43.3)	13 (56.7)	
BMI (kg/m^2^)				*n.s* > .05
>24.9	25	14 (56)	11 (44)	
≤24.9	33	15 (45.5)	18 (54.5)	
Number of pregnancy (time)				*n.s* > .05
≥3	22	12 (54.5)	10 (45.5)	
<3	36	17 (47.2)	19 (52.8)	
Previous history of thrombosis				*p* < .01
Yes	13	10 (76.9)	3 (23.1)	
No	45	19 (42.2)	26 (57.8)	
History of adverse pregnancy delivery				*p* < .01
<10 weeks abortion (1 time)	11	6 (54.5)	5 (45.5)	
<10 weeks abortion (2 times)	9	6 (66.7)	3 (33.3)	
<10 weeks abortion (≥3 consecutive times)	3	2 (66.7)	1 (33.3)	
≥10 weeks stillbirth	10	7 (70)	3 (30)	
<34 weeks delivery	6	4 (66.7)	2 (33.3)	
Late pre‐eclampsia/late preterm birth (34–36 weeks gestation)	2	2 (100)	0 (0)	
No history of adverse pregnancy delivery	17	2 (11.8)	15 (88.2)	

*Note*: *p* < .05 represents significant difference.

### 3.2 Anti‐B_2_GPI, ACA, MMP‐9, iNOS, ICAM‐1, and MCP‐1 expressions in APS

**Figure 2:**
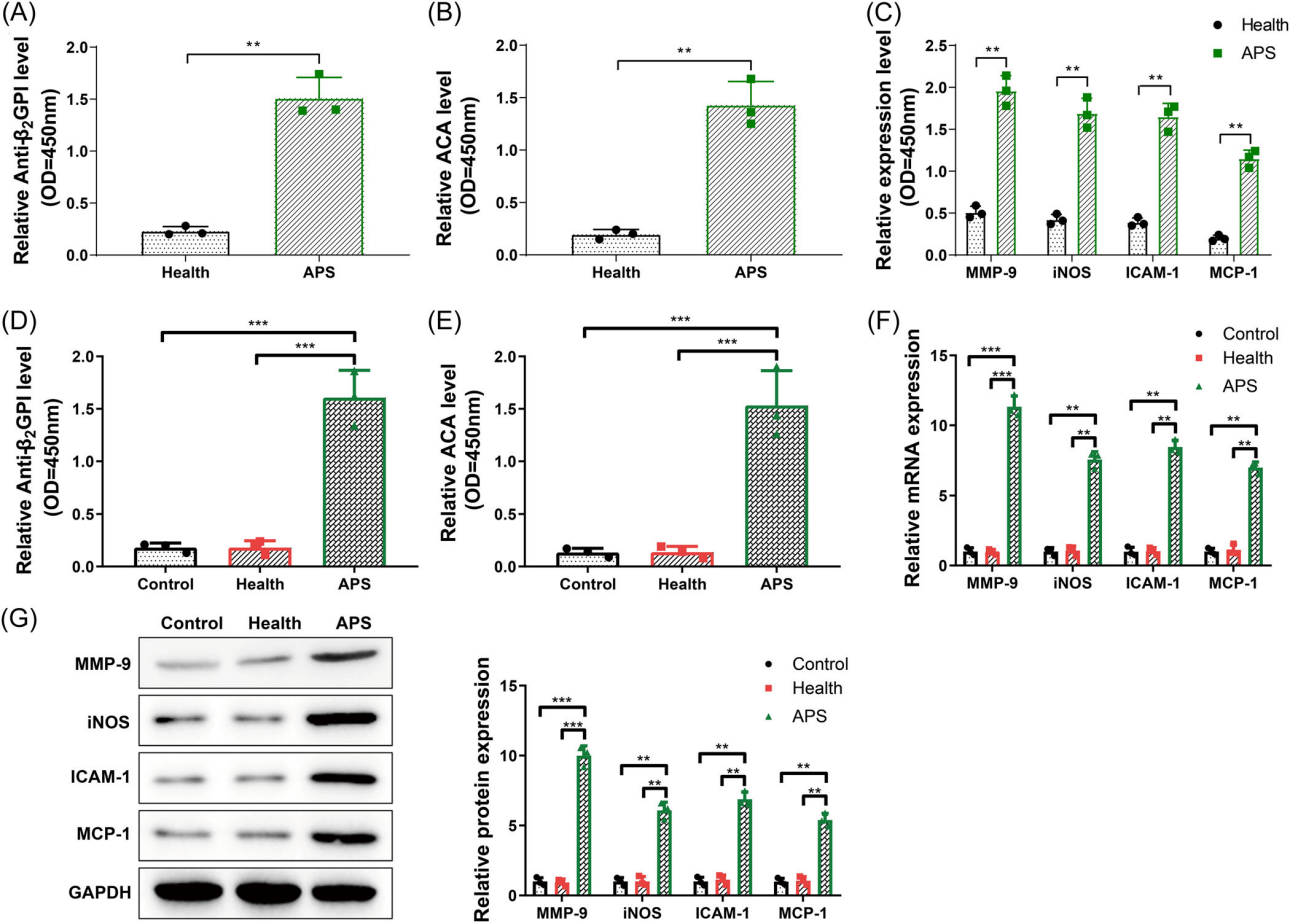
APS increased anti‐B_2_GPI and ACA levels and upregulated MMP‐9, iNOS, ICAM‐1, and MCP‐1 expressions. (A) Anti‐B_2_GPI level in peripheral blood serum of APS patients (*N* = 58) and healthy women (*N* = 46) detected by ELISA; (B) ACA level in peripheral blood serum of APS patients (*N* = 58) and healthy women (*N* = 46) detected by ELISA; (C) The expressions of MMP‐9, iNOS, ICAM‐1, and MCP‐1 protein in serum of APS patients (*N* = 58) and healthy women (*N* = 46) detected by ELISA; (D) Anti‐B_2_GPI level in HUVEC culture medium of different treatment groups detected by ELISA; (E) ACA level in HUVEC culture medium of different treatment groups detected by ELISA; (F) The mRNA expressions of MMP‐9, iNOS, ICAM‐1, and MCP‐1 in HUVECs of different treatment groups detected by qRT‐PCR; (G) The protein expressions of MMP‐9, iNOS, ICAM‐1, and MCP‐1 in HUVECs of different treatment groups detected by western blot. The experiment was repeated three times. *T* test was used for comparison between two groups, one‐way analysis of variance test for comparisons among multiple groups, and Tukey's multiple comparisons test for post hoc multiple comparisons. *p < .05, **p < .01, ***p < .001. ACA, anticardiolipin antibody; APS, antiphospholipid syndrome.

Multiple studies have indicated that anti‐B_2_GPI and ACA participate in the pathogenesis of APS through inflammatory factor production, endothelial cell and platelet activation, and coagulation factor and complement activation.^9^, ^20^, ^21^, ^22^ Therefore, we first analyzed the expressions of anti‐B_2_GPI, ACA, and related factors in the peripheral serum of 58 APS patients and 46 healthy women by performing ELISA. The positive rates of anti‐B_2_GPI and ACA and the levels of MMP‐9, iNOS, ICAM‐1, and MCP‐1 in the serum of APS patients were higher than those of healthy women (Figure 2A–C, *p* < .05). The positive rates of anti‐B_2_GPI and ACA in the cell culture medium of the control group, the health group and the APS group were further tested by ELISA, through which we observed that the positive rates of anti‐B_2_GPI and ACA in the APS group were higher than those in the health group and the control group (Figure 2D,E, *p* < .05). In addition, the mRNA and protein expressions of MMP‐9, iNOS, ICAM‐1, and MCP‐1 in HUVECs of the control group, the health group and the APS group were detected by qRT‐PCR and western blot. Here, we observed that the mRNA and protein expressions of MMP‐9, iNOS, ICAM‐1, and MCP‐1 in the APS group were markedly higher than those in the health group and the control group (Figure 2F,G, *p* < .05). The above results demonstrated that APS increased anti‐B_2_GPI and ACA positive rates and elevated the expressions of MMP‐9, iNOS, ICAM‐1, and MCP‐1.

### 3.3 Effects of APS on HUVEC senescence and biological behaviors

**Figure 3:**
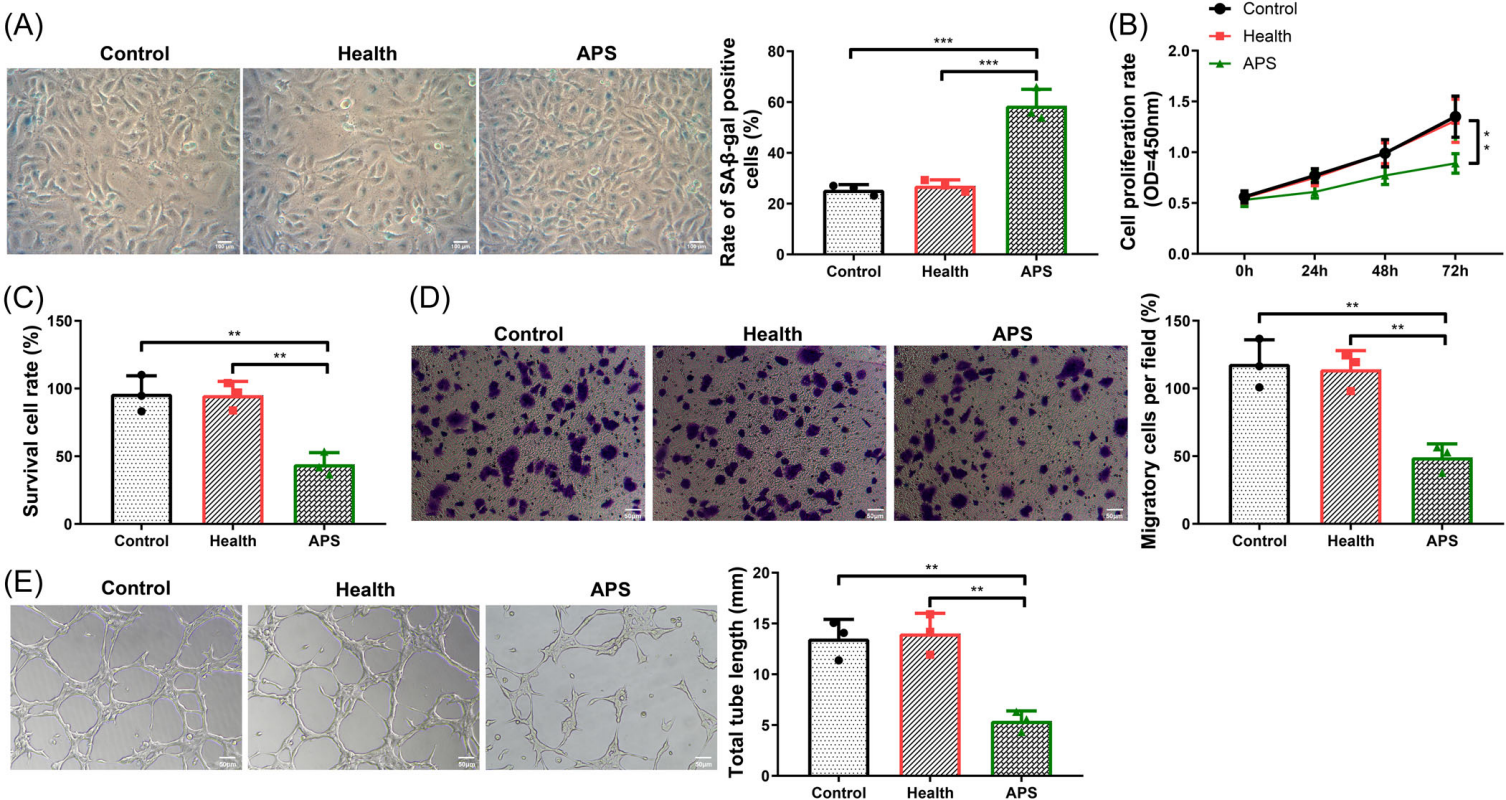
APS affected HUVEC senescence and biological behaviors. (A) SA‐β‐gal staining (positive in blue) assay for the detection of HUVEC senescence in different treatment groups; (B) CCK‐8 assay for the detection of HUVEC proliferation in different treatment groups; (C) trypan blue staining for the detection of HUVEC viability in different treatment groups; (D) transwell assay for the detection of HUVEC migration in different treatment groups; (E) matrigel tube formation assay for the detection of HUVEC angiogenesis in different treatment groups. The experiment was repeated three times. *T* test was used for comparisons between two groups, one‐way analysis of variance test for comparisons among multiple groups, and Tukey's multiple comparisons test for post hoc multiple comparisons. *p < .05, **p < .01, ***p < .001. APS, antiphospholipid syndrome.

Cell senescence was determined by SA‐β‐gal staining (positive in blue), and the results showed that the positive rate of SA‐β‐gal in the APS group was higher than that in the health and control groups (Figure 3A, *p* < .05). The cell proliferation and cell viability of each group were detected by CCK‐8 and trypan blue staining, respectively. The results illustrated that the cell proliferation and cell viability of the APS group were lower relative to the health and control groups (Figure 3B,C, *p* < .05). Through transwell assay, we observed that the number of migrating cells in the APS group was markedly reduced relative to the health and control groups (Figure 3D, *p* < .05). Through matrigel tube formation assay, we also found that the angiogenesis ability of the APS group was lower relative to the health group (Figure 3E, *p* < .05). The above results indicated that APS promoted HUVEC senescence and reduced cell proliferation ability, cell viability, migration as well as angiogenesis ability.

### 3.4 Overexpression of SAMD1 reversed the effects caused by APS

**Figure 4:**
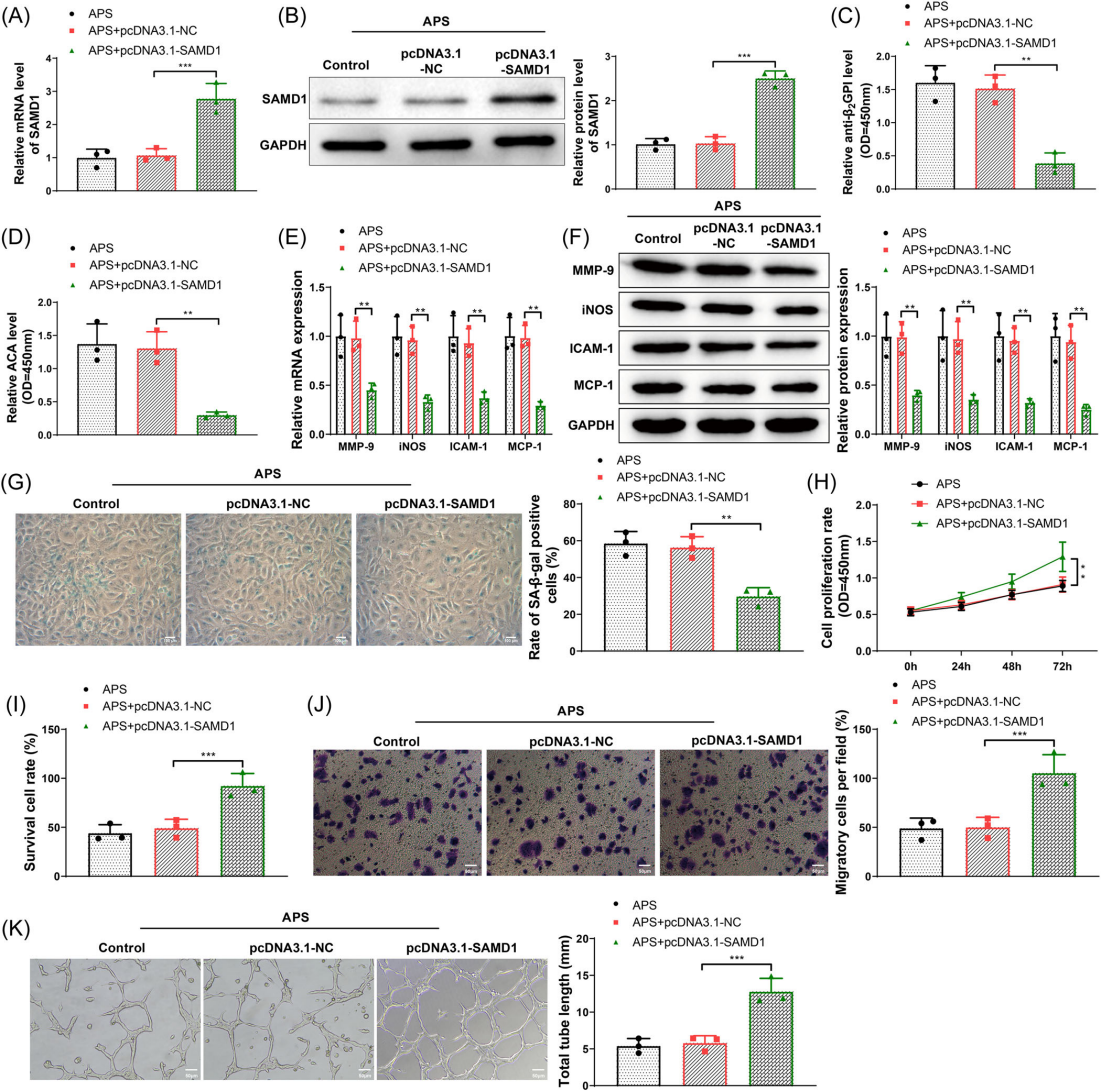
Overexpression of SAMD1 reversed the effects caused by APS. (A and B) qRT‐PCR or western blot for the detection of the expression of SAMD1 in HUVECs of different treatment groups; (C) ELISA for the detection of anti‐B_2_GPI level in HUVEC culture medium of different treatment groups; (D) ELISA for the detection of ACA level in HUVEC culture medium of different treatment groups; (E) qRT‐PCR for the detection of expressions of MMP‐9, iNOS, ICAM‐1, and MCP‐1 mRNA in HUVECs of different treatment groups; (F) western blot for the detection of expressions of MMP‐9, iNOS, ICAM‐1, and MCP‐1 protein in HUVECs of different treatment groups; (G) SA‐β‐gal staining (positive in blue) for the detection of senescence of HUVECs of different treatment groups; (H) CCK‐8 assay for the detection of proliferation ability of HUVECs of different treatment groups; (I) trypan blue staining for the detection of viability of HUVECs of different treatment groups; (J) transwell assay for the detection of migration ability of HUVECs of different treatment groups; (K) matrigel tube formation assay for the detection of angiogenic ability of different treatment groups. The experiment was repeated three times. One‐way analysis of variance test was used for comparisons among multiple groups, and Tukey's multiple comparisons test for post hoc multiple comparisons. *p < .05, **p < .01, ***p < .001. ACA, anticardiolipin antibody; APS, antiphospholipid syndrome.

To investigate the effects of SAMD1 on APS‐induced vascular injury and pregnancy complications, we tested various parameters of HUVECs in the APS, pcDNA3.1‐NC, and pcDNA3.1‐SAMD1 groups. The expression of SAMD1 in HUVECs of each group was detected by qRT‐PCR, and the results revealed that the expression of SAMD1 in the pcDNA3.1‐SAMD1 group was higher than that in the pcDNA3.1‐NC group (Figure 4A,B, *p* < .05), indicating that the cell transfection effect was good. The levels of anti‐B_2_GPI and ACA in the cell culture medium of the APS, pcDNA3.1‐NC and pcDNA3.1‐SAMD1 groups were detected by ELISA. The observation indicated that the levels of anti‐B_2_GPI and ACA in the pcDNA3.1‐SAMD1 group were markedly lower relative to the pcDNA3.1‐NC group (Figure 4C,D, *p* < .05), and the levels of anti‐B_2_GPI and ACA in the pcDNA3.1‐NC group were not significantly different from those in the APS group. The results of qRT‐PCR and western blot for measuring the mRNA and protein expressions of MMP‐9, iNOS, ICAM‐1, and MCP‐1 in HUVECs in the APS, pcDNA3.1‐NC, and pcDNA3.1‐SAMD1 groups showed that the mRNA and protein expressions of MMP‐9, iNOS, ICAM‐1, and MCP‐1 in HUVECs in the pcDNA3.1‐SAMD1 group were also significantly lower than those in the pcDNA3.1‐NC group (Figure 4E,F, *p* < .05). The above results demonstrated that overexpression of SAMD1 was able to reverse the APS‐induced elevation of anti‐B_2_GPI and ACA levels as well as MMP‐9, iNOS, ICAM‐1, and MCP‐1 expressions.

Cell senescence was then determined by SA‐β‐gal staining (positive in blue). The results exhibited that the positive rate of SA‐β‐gal in the pcDNA3.1‐SAMD1 group was lower versus the pcDNA3.1‐NC group (Figure 4G, *p* < .05), and there was no obvious difference between the pcDNA3.1‐NC group and the APS group. The cell proliferation ability and cell viability of HUVECs in the three groups were detected by CCK‐8 assay and trypan blue staining, respectively. The results illustrated that the cell proliferation and cell viability in the pcDNA3.1‐SAMD1 group were markedly higher relative to the pcDNA3.1‐NC group (Figure 4H,I, *p* < .05), and there was no significant difference between the pcDNA3.1‐NC group and the APS group. The transwell assay demonstrated that the number of migrating cells in the pcDNA3.1‐SAMD1 group was increased versus the pcDNA3.1‐NC group (Figure 4J, *p* < .05), and no marked difference was seen between the pcDNA3.1‐NC group and the APS group. The matrigel tube formation assay presented that the angiogenic ability of the pcDNA3.1‐SAMD1 group was enhanced relative to the pcDNA3.1‐NC group (Figure 4K, *p* < .05), and no clear difference was found between the pcDNA3.1‐NC group and the APS group. In addition, transfection of pcDNA3.1‐SAMD1 into HUVECs in the control group significantly increased the expression of SAMD1 (Supporting Information: Figure 1A,B, *p* < .05), but did not change the levels of anti‐B_2_GPI and ACA, cell proliferation ability, and cell viability (Supporting Information: Figure 1C–F), which suggested that the effect of overexpressed SAMD1 seemed to be specific to APS. In summary, overexpression of SAMD1 was able to reverse the effects caused by APS.

### 3.5 Overexpression of SAMD1 reversed APS‐induced pregnancy complications in pregnant APS mouse model

**Figure 5:**
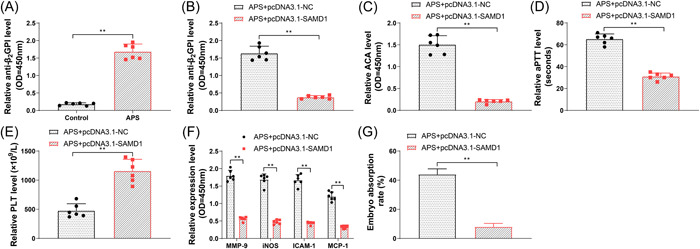
Overexpression of SAMD1 reversed APS‐induced pregnancy complications in pregnant APS mouse model. (A) ELISA for the detection of expression of anti‐B_2_GPI in the serum of mice in the control group and APS experimental group; (B) ELISA for the detection of anti‐B_2_GPI level in the serum of pregnant mice in the pcDNA3.1‐NC group and pcDNA3.1‐SAMD1 group; (C) ELISA for the detection of ACA level in the serum of pregnant mice in the pcDNA3.1‐NC group and pcDNA3.1‐SAMD1 group; (D) aPTT detection kit for the detection of aPTT level in the serum of pregnant mice in the pcDNA3.1‐NC group and pcDNA3.1‐SAMD1 group; (E) PLT detected by automatic blood cell analyzer; (F) ELISA for the detection of expressions of MMP‐9, iNOS, ICAM‐1, and MCP‐1 in the serum of pregnant mice in the pcDNA3.1‐NC group and pcDNA3.1‐SAMD1 group; (G) after the pregnant mice were killed, the embryo absorption rate was detected. *N* = 6, and three biological replicates were set for each sample. *T* test was used for comparison between two groups, and Tukey's multiple comparisons test was used for post hoc multiple comparisons. *p < .05, **p < .01, ***p < .001. ACA, anticardiolipin antibody; APS, antiphospholipid syndrome.

To further validate the mechanism of action of SAMD1, APS pregnant mouse models were constructed. The anti‐B_2_GPI levels in the serum of the mouse models in the control group and APS group were tested by ELISA, and the observation revealed that the anti‐B_2_GPI levels in the APS group were markedly higher relative to the control group (Figure 5A, *p* < .05), indicating that the APS mouse model was successfully constructed. Next, the serum levels of anti‐B_2_GPI, ACA, aPTT, and PLT in the pcDNA3.1‐SAMD1 and pcDNA3.1‐NC groups were detected. The results showed that the serum levels of anti‐B_2_GPI, ACA, and aPTT in the pcDNA3.1‐SAMD1 group were significantly lower than those in the pcDNA3.1‐NC group (Figure 5B–D, *p* < .05), and the PLT level was significantly higher than that in the pcDNA3.1‐NC group (Figure 5E, *p* < .05). The serum levels of MMP‐9, iNOS, ICAM‐1, and MCP‐1 in the pcDNA3.1‐NC group and the pcDNA3.1‐SAMD1 group were measured by ELISA, and it was found that the serum levels of MMP‐9, iNOS, ICAM‐1, and MCP‐1 in the pcDNA3.1‐SAMD1 group were significantly lower than those in the pcDNA3.1‐NC group (Figure 5F, *p* < .05). The above results showed that overexpression of SAMD1 could reduce anti‐B_2_GPI and ACA levels, shorten aPTT, and increase PLT, thereby reversing APS‐induced vascular injury to some extent. In addition, we calculated the embryo absorption rate in the pcDNA3.1‐NC group and the pcDNA3.1‐SAMD1 group after isolation of mouse embryos, and the results revealed that APS would induce pregnancy complications such as unexplained fetal death and abortion during pregnancy. However, the embryo resorption rate of mice in the pcDNA3.1‐SAMD1 group was significantly lower than that in the pcDNA3.1‐NC group (Figure 5G, *p* < .05). The above results indicated that overexpression of SAMD1 was able to effectively reduce the fetal loss rate in APS mice.

## 4 DISCUSSION

APS is a multisystem autoimmune disease that is mainly caused by upregulation of aPL in serum.^23^ By acting on the membrane phospholipids of placental vascular endothelial cells, aPL interferes with the release of thrombomodulin and plasminogen activator, leading to hypercoagulability and change of vascular endothelium pathological mechanisms which consequently result in thrombosis and adverse events in pregnancy. Based on the GEO database, we found that SAMD1 expression was decreased in serum from APS patients compared with that from healthy control women. In this study, we found SAMD1 was related with APS‐induced pregnancy complications.

First, we found that the positive rates of anti‐B_2_GPI and ACA and the expressions of MMP‐9, iNOS, ICAM‐1, and MCP‐1 were elevated in APS. Endothelial dysfunction is an important driver of thrombosis in APS, which is affected through autoimmunity and inflammation pathways.^24^ MMP‐9, iNOS, ICAM‐1, and MCP‐1 were demonstrated to participate in the progression of inflammatory and immune disease.^25^, ^26^, ^27^ A previous in vitro study also found that aPL could suppress HUVEC proliferation, migration, and angiogenesis.^28^ In line with the established evidence, our further experiments indicated that APS promoted HUVEC senescence and reduced cell proliferation, cell viability, migration as well as angiogenesis ability.

In this study, we found that the expression of SAMD1 was downregulated in APS. Overexpression of SAMD1 was able to reverse APS‐induced elevation of anti‐B_2_GPI and ACA levels as well as MMP‐9, iNOS, ICAM‐1, and MCP‐1 expressions. Further experiments also showed that overexpression of SAMD1 promoted proliferation ability, viability, migration as well as angiogenesis ability in HUVECs. The above findings indicated that overexpression of SAMD1 could reverse the effects caused by APS. SAMD1 has been identified as a transcriptional repressor which acts at unmethylated CGIs to regulate mouse embryonic stem cell differentiation processes,^29^ but the specific function of SAMD1 in human diseases is barely known. A previous study revealed that SAMD9 could influence cytokine expression and T cell proliferation and act as an anti‐inflammatory factor in rheumatoid arthritis.^30^ It was reported that human SAMD4A could serve as a newly identified breast tumor angiogenesis inhibitor through regulating the balance of angiogenesis program.^31^ More importantly, it was observed that loss of SAMD1 decreased the levels of some metabolism and angiogenesis‐related factors, thereby causing failure of blood vessel maturation in mice.^32^ Those studies on the role of other SAMD proteins in inflammation and angiogenesis might provide supportive evidence for our findings about SAMD1. Accumulating studies have provided the evidence that APS might cause recurrent early abortion, fetal loss, or other adverse events during pregnancy.^3^, ^8^ To further validate the mechanism of action of SAMD1, APS pregnant mouse model was constructed. We found that overexpression of SAMD1 could reduce anti‐B_2_GPI and ACA levels, shorten aPTT, and increase PLT, thereby reversing APS‐induced vascular injury to some extent. Additionally, overexpression of SAMD1 was able to effectively reduce the fetal loss rate in APS mice.

Taken together, we found that SAMD1 could inhibit cellular senescence, promote cell proliferation, cell viability, migration ability and angiogenesis, and effectively reduce APS‐induced embryo loss. However, our study has some limitations which need to be solved in future studies. First, in this study, we only explored the relationship between SAMD1 and APS, and failed to investigate up/downstream pathways and related mechanism. APS is a contributor for various adverse events related with pregnancy, but we only investigated the fetal loss rate in a pregnant mouse model of APS. Nonetheless, our conclusion offers a possible rationale to target SAMD1 for the diagnosis and treatment of APS.

## AUTHOR CONTRIBUTIONS

Ran An and Peiling Li conceived the ideas. Ran An, Yanqi Yang and Lei Liu designed the experiments. Ran An, Yanqi Yang, and Lei Liu analyzed the data. Ran An and Peiling Li provided critical materials. Ran An wrote the manuscript. Peiling Li supervised the study. All the authors have read and approved the final version for publication.

## CONFLICT OF INTEREST STATEMENT

The authors declare no conflict of interest.

## Supporting information

Supplementary information.

## Data Availability

The data sets used or analyzed during the current study are available from the corresponding author on reasonable request.
