# Supplementary figures and images for: SAMD1 attenuates antiphospholipid syndrome‐induced pregnancy complications
Source: Immun Inflamm Dis. 2023 Oct 30;11(10):e1006. doi: 10.1002/iid3.1006 (PMC10614121; doi:10.1002/iid3.1006)

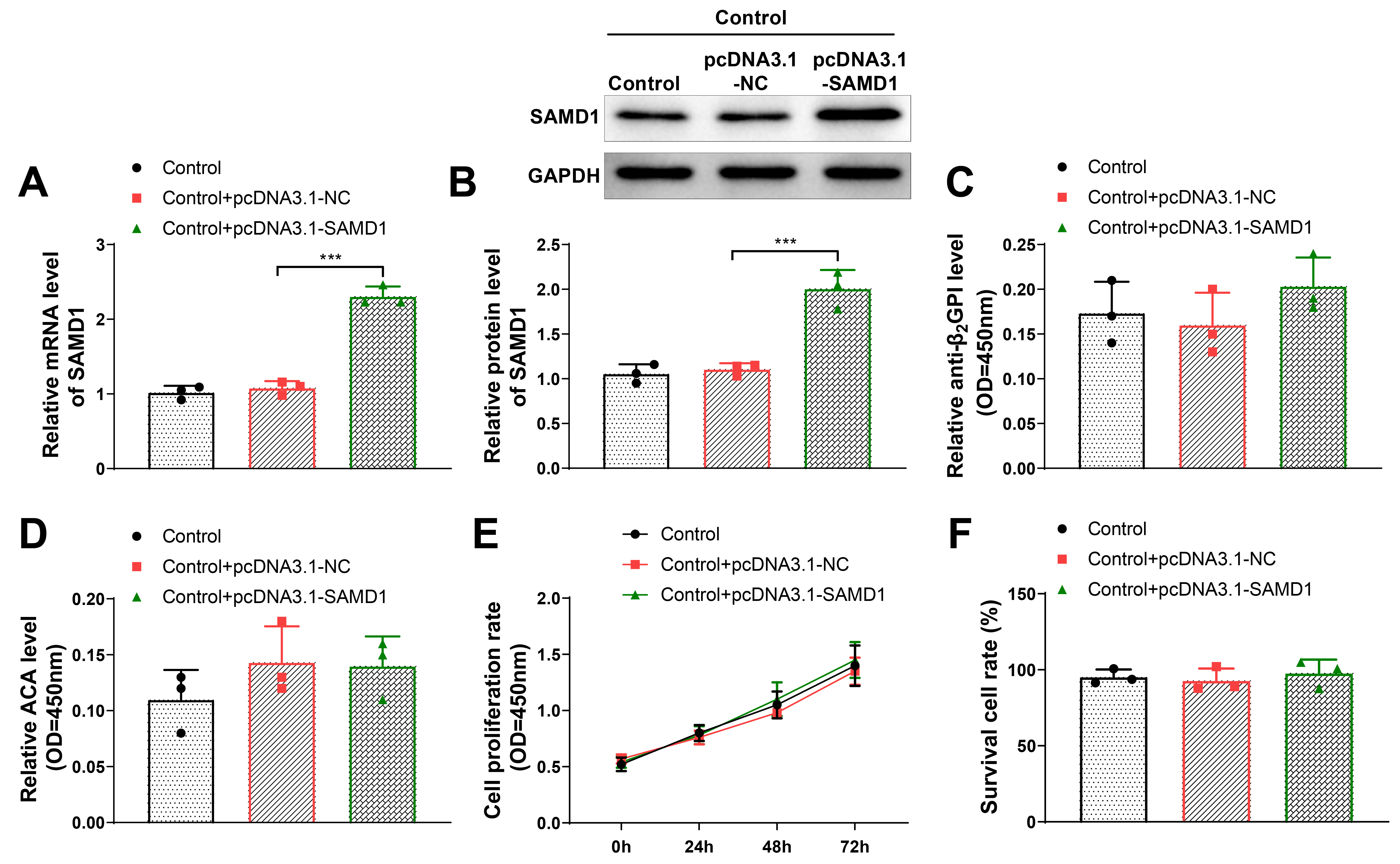

Supplement: Supplementary file 1 — Supplementary information. [file IID3-11-e1006-s001.tif]
